# Valuing the impact of self-rated health and instrumental support on life satisfaction among the chinese population

**DOI:** 10.1186/s12889-022-13626-7

**Published:** 2022-06-20

**Authors:** Chee Hon Chan, Anna Wong

**Affiliations:** 1 Department of Applied Social Sciences, The Hong Kong Polytechnic University, Hung Hom, Hong Kong; 2 Centre for Social Policy and Social Entrepreneurship, The Hong Kong Polytechnic University, Hung Hom, Hong Kong; 3 The HKJC Centre for Suicide Research and Prevention, The University of Hong Kong, Pok Fu Lam, Hong Kong

**Keywords:** Wellbeing valuation, Self-rated health, Instrumental support, Willingness-to-pay, Chinese

## Abstract

**Background:**

Research has highlighted that satisfaction in health, and instrumental support (IS) are key areas of life affecting an individual’s wellbeing. Many social and public health initiatives use these two intervention mechanisms to improve individual’s wellbeing. For the purpose of cost-benefit assessment, there has been growing interest in expressing these intervention effects in economic terms. However, only a handful of studies have ever estimated these effects in economic terms, none of which examined them in a Chinese context. The aim of this study is to extend this line of valuation work to the Chinese population, estimating the implicit willingness-to-pays on the effects of improving individuals’ self-rated health (SRH) status and IS on their life satisfaction (LS).

**Methods:**

Using data from a two-wave representative panel survey in Hong Kong (n = 1,109), this study conducted a cross-lagged analysis with a structural equation modelling technique to examine the causal effects of SRH and IS on LS. The use of this cross-lagged approach was an effort to minimise the endogeneity problem. Then, substituting the respective estimates to the formulae of compensating surplus, the marginal rate of substitution of SRH and IS with respect to individual’s equivalised monthly household income (HI) were estimated and were then expressed as the implicit willingness-to-pays on the effect of improving individuals’ SRH and IS on their LS.

**Results:**

The cross-lagged analysis ascertained the causal effects of SRH (β = 0.074, 95% Confidence Interval: 0.021, 0.127) and IS (β = 0.107, 95% Confidence Interval: 0.042, 0.171) on individuals’ satisfaction with life. Translating into the concept of compensating surplus, the implicit monetary values of improving the sample’s SRH from “poor health” to “excellent health” and their perceived IS from “little support” to “a lot of support” are equivalent to an increase in their equivalised monthly HI by US$1,536 and US$1,523 respectively.

**Conclusions:**

This study is the first to derive the implicit monetary values of SRH and IS on individual’s LS in a predominantly Chinese society, and it has implications for the cost-benefit assessment in wellbeing initiatives within the population.

**Supplementary Information:**

The online version contains supplementary material available at 10.1186/s12889-022-13626-7.

## Background

Individuals’ satisfaction with life has been conceptualised as one of the major indicators of one’s overall wellbeing [1, 2], and a substantial body of research from the domains of life literature has postulated that individuals’ wellbeing depends on their appraisals of various aspects of daily life activities [3, 4]. Previous research has highlighted both physical health and social relationships as key factors that determine individuals’ psychological experience and assessment of life [5, 6]. The research is supported by Engel’s biopsychosocial model, which conceptualises a person’s biological, psychological, and social domains as distinct systems that causally influence each other and together contribute to one’s health and wellbeing [7]. For example, impaired physical health is associated with a decline in positive affect and an increase in negative feelings, such as a depressed mood and anger [8]. With worsening physical and mental health, individuals’ involvement in social activities is likely to be restricted, resulting in limited social connection [9, 10]. Research has shown that social relationships have a powerful influence on physical health and longevity [11]. Individuals having greater social participation and support from their social circle also tended to have a higher level of life satisfaction (LS) [12].

Social support has been theorised as having emotional and instrumental dimensions [13]. The emotional dimension, usually known as emotional support, refers to the expressions of love and care. The instrumental dimension comprises of several types of support, including tangible aid, such as labour and finances, and informational support, the sharing of useful information for solving stress-inducing issues [14, 15]. Although with exceptions [16, 17], previous studies have repeatedly highlighted that instrumental support offers practical solutions and also signify emotional care to the recipients [18]. Instrumental support (IS) can serve as a buffer to adverse psychological impacts caused by stressors in life [13, 19]. It has been found to alleviate the burden of depressive symptoms, generate happiness and boost overall LS [20, 21]. Research has pointed out that these factors, e.g., health and social relationships, often interact with each other in influencing individuals’ wellbeing. For instance, healthier individuals are more likely to participate in social activities and perceive having greater support from their families and friends [22].

Over the years, policy thinkers have continuously emphasised a key objective of health and social policy is to improve individuals’ wellbeing and collectively raise population welfare [23–26]. The concept of individuals’ wellbeing has now taken centre stage in many public policy discussions. Worldwide, initiatives to enhance population wellbeing have been growing, such as enhancing access to health care and building social support and community cohesion. This is no different within Chinese and/or East-Asian populations, where efforts are being made to assimilate research from Western societies while ensuring it is culturally appropriate. Interest in the cost-effectiveness of wellbeing initiatives has also been stacking up. However, assessing the cost-effectiveness of wellbeing initiatives can be challenging since the impacts of many of these initiatives, such as improving health status and social support, are difficult to measure in economic terms (i.e., non-market goods without direct monetary value). Prior research has used the stated-preference valuation methods to resolve this challenge, which typically required individuals to answer hypothetical questions in order to elicit their implicit willingness-to-pay, or the ‘shadow price’, on the non-market social goods [27]. However, a growing body of research has cautioned the use of these valuation methods since the cognitive biases caused by the experimental settings could affect the valuation decision and hence leads to inadequate estimates [28–32].

### Subjective wellbeing valuation

Against this backdrop, recent research has developed an alternative method to value non-market goods, which is often referred to as the subjective wellbeing valuation [30, 33, 34]. Typically, these valuation methods will make use of individuals’ subjective wellbeing data, such as life satisfaction (LS), collected through large-scale surveys. In this context, measures of LS that capture individuals’ appraisals of their overall quality of life with regards to past experience, expectations of the future, and comparison to others [35], are considered to be indicators of one’s experienced utility [36]. Surveys that explore how exposure to some non-market circumstances could causally affect individuals’ LS are then interpreted as a direct empirical approximation of how the goods of interest alter individual welfare (see Additional file 1 for a concept illustration). In other words, it correlates the non-market goods (e.g., health status) with individuals’ LS and evaluates them directly in relation to the effect of income on LS [37]. These methods create the implicit “trade-offs” between income and non-market goods of interest, which can then be interpreted as the willingness-to-pay of the goods of interest (i.e., additional income to pay or accept in order to compensate for the changes in satisfaction with life for the losses or gains in some particular conditions) [38]. Scholars have highlighted that this wellbeing valuation approach does not rest upon individuals’ decisions in the valuation process, hence avoiding potential cognitive biases that could have been involved in the stated-preference methods [38, 39].

The use of this wellbeing valuation approach has gained considerable traction in recent years and has started to be applied in estimating the economic term for various health and social conditions. Internationally, only a handful of studies have used this wellbeing valuation approach to estimate the effects of improving self-rated health (SRH) status and social relations on LS [39–44]. However, the estimates derived from these studies varied considerably. For instance, a study in the United Kingdom estimated that the economic value of improving an individual’s SRH from ‘poor health’ to ‘excellent health’ is equivalent to increasing his/her annual HI per capita £303,000 [40], other studies reported a substantially smaller estimate (e.g., US$1,644 - US$1,692 [43]; see Additional file 2). Some variations of the estimates may be methodologically related as some of the prior work ignored the endogeneity issue in their estimation [40, 44, 45], which is a methodological concern that could introduce biases and cause an overestimation of the monetary value of the non-market goods [45, 46]. The cultural difference may also come into play. Prior research has highlighted that people from different cultures weigh domains of life differently when they consider their general LS [47]. For instance, the linkage between income and LS has been reported to be stronger in societies upholding greater value on individualism, as a higher income symbolises greater personal success [48]. Whereas in cultures with a greater influence from Confucianism and Collectivism (e.g., Chinese and some other Southeast Asian societies), individuals’ appraisal of life is found to have a greater dependence on the external perception of their social groups, such as members of the family and friends [49]. All these indicate that estimations of implicit willingness-to-pays of non-market social goods, e.g. SRH and social relationship, should be sociocultural-bounded.

### Motivation of this study

This study was motivated by the relatively few applications of the subjective wellbeing valuation on non-market social goods in the Chinese context, despite fast-growing policy attention on individuals’ wellbeing in this region [50–53]. As pointed out earlier, many of these existing valuation works were conducted in the Western setting, and their findings may not be directly generalisable to other socio-cultural settings. Only two studies have used this approach to estimate the effects of improving health status and social relationships on LS in an Asian context [43, 44], and none of which examined it in a Chinese context. It limits the use of cost-benefit analysis to assess the cost-effectiveness of wellbeing initiatives in the region.

Using a recently conducted two-wave population household survey in Hong Kong, the aim of this study was to estimate the implicit willingness-to-pay of the effects of enhancing individuals’ SRH and perceived support from family and friends on their satisfaction with life in the Chinese population. This study focused on these two factors as they are intervention mechanisms commonly used to enhance individual welfare; deriving the monetary value of these two variables provides wide application in cost-benefit policy analysis. This study hypothesised that individuals’ SRH, IS, and HI are all causally associated with their LS, as repeatedly highlighted in prior research [39, 43]. While this study conducted a cross-lagged analysis to ascertain these propositions, the main contribution of this work is on estimating the implicit willing-to-pays of SRH and IS among the Chinese population.

## Methods

### Sample

Data used in this study were extracted from the Hong Kong Panel Survey for Poverty Alleviation, which is a two-wave household survey with a representative sample recruited through a stratified random sampling strategy by 412 geographical constituency areas (i.e., District Constituency Areas) [54].^1^ The panel survey involved conducting face-to-face interviews with the household head to elicit a variety of information; each interview lasted approximately 60 min. Content of the survey and details of the sampling strategy has been reported elsewhere [55]. The first wave of the panel survey was conducted between September 2015 and April 2016, in which 2,002 households were recruited. These households were interviewed again 12 months after the first interview (the second wave), and 1,109 households were retained (retention rate: 55.4%).

### Measurement

This study retrieved data on the sample’s socioeconomic and demographic characteristics (i.e., age, sex, educational attainment, marital status, employment status, housing tenure, household size, and monthly HI), and their status of SRH, perceived IS from the sample’s social network and satisfaction with life. The survey assessed the sample’s SRH by a single item eliciting their overall health condition (i.e., In general, would you say your health is (1) poor, fair, good, very good, or (5) excellent?). This one-item measure of SRH status is widely used in health research [56], and previous studies have shown that this is a valid and reliable measure of one’s overall physical and mental health condition [57]. Two earlier wellbeing valuation studies also used this measure to capture an individual’s perceived health status [39, 40].

Similar to previous studies using a few items inquiring about an individual’s perceived level of tangible and informational assistance from their social circles [58], this survey used three items to measure the sample’s IS from family, friends, and other available sources. In particular, one item explored on sample’s perceived financial support (i.e., How much help would you receive if you need money for an emergency situation?), and another examined the availability of physical assistance (i.e., How much help would you receive when you need physical help and need someone to take care of you?*)*. The third item focused on the informational resources (i.e., How much help would you receive when you need someone’s advice in making important decisions?). According to the literature, these tangible supports would also convey emotional care. Respondents were asked to assess their perceived level of support ranging from (1) no support at all to (4) a lot of support, and the total score hence ranges between 3 and 12. A post-hoc evaluation suggested that Cronbach’s alpha estimates of these three items were 0.79 in both the 1st wave and the 2nd wave, respectively.

For the sample’s satisfaction with life, the five-item Chinese version of the Satisfaction with Life Scale (SWLS) was employed. The total scores of the SWLS range from 5 (extremely dissatisfied) to 35 (extremely satisfied). The Chinese version of the SWLS has been validated and was found to have adequate validity and reliability [59]. The Cronbach’s alpha estimates of the SWLS were 0.86 in the 1st wave and 0.88 in the 2nd wave, respectively.

### Statistical analyses

Analysis in wellbeing valuation work can be generally conceptualised as having two-part. The first part involves a regression analysis estimating the effects of some non-market conditions (i.e., SRH and IS) on LS and, respectively, the effect of an economic indicator (e.g., HI) on LS. Estimates of these effects would then form the marginal rate of substitution of the non-market conditions with respect to the economic indicator. As mentioned earlier, one major methodological concern that could introduce biases in this part of estimation is the endogeneity issue [60] and failing to account for it can cause an overestimation of the monetary value of the non-market goods [45, 46]. Studies typically used instrumental variables to handle the problem, but the use of the instruments also has its own constraints. Specifically, variables that qualify as an instrument should fulfil the independence assumption, i.e., the instruments should not share causes with the outcome variables (e.g., LS). Researchers have highlighted that it is very difficult to identify appropriate instruments that meet this assumption [45], as “almost every factor determines LS” [61].

**Fig. 1:**
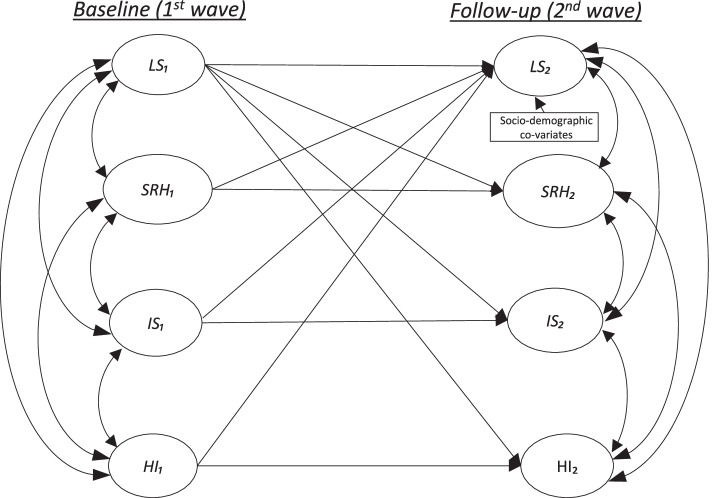
Conceptual illustration of the cross-lagged analysis of the relationships between SRH, IS, HI, LS, and socio-demographic covariates

An alternative way to tackle the endogeneity issue in wellbeing valuation is the use of a cross-lagged analysis [62]. Figure 1 shows a conceptual model of a cross-lagged analysis applying in an examination of the relationships between SRH, IS, HI, LS, and socio-demographic covariates. As shown, the model specified three main types of effects: one reflects the temporal autoregressive effects of the variable of SRH, IS, HI, on LS (e.g., ${\beta }_{a}$: the causal effects of ${\text{S}\text{R}\text{H}}_{1}$ at baseline on ${\text{S}\text{R}\text{H}}_{2}$ at follow-up) and another type is the cross-sectional correlations among SRH, IS, HI and LS, (e.g., ${\beta }_{cor}$: the correlations between ${\text{S}\text{R}\text{H}}_{1}$ and ${\text{L}\text{S}}_{1}$ at baseline). The remaining type of effects examine the reciprocal nature between SRH, IS, HI, and LS (e.g., 𝛽c: the causal effect of ${\text{S}\text{R}\text{H}}_{1}$ and ${\text{I}\text{S}}_{1}$ at baseline on ${\text{L}\text{S}}_{2}$ at follow-up and, 𝛽cry: the reverse causal effect of ${\text{L}\text{S}}_{1}$ at baseline on ${\text{S}\text{R}\text{H}}_{2}$ and ${\text{I}\text{S}}_{2}$at follow-up).

In the context of this valuation work, the main subject of interest is the 𝛽c (e.g., the causal effect of ${\text{S}\text{R}\text{H}}_{1}$ on ${\text{L}\text{S}}_{2}$), as it will be used for calculating the marginal rate of substitution. Specifications of the other five effects are ways to reduce the endogeneity problem in the estimation process. Specifically, given the endogeneity bias typically arises from reverse causality (𝛽rc) and omitted influences on the dependent variable (e.g., temporal autoregressions, ${\beta }_{a}$,and cross-sectional correlations, ${\beta }_{cor}$) [60], the cross-lagged model explicitly addressing them help to reduce these potential influences in the estimation of the causal effect of SRH on LS. The cross-lagged model is computed based on a structural equation modelling technique, which is a form of simultaneous regression technique often used in the work of subjective wellbeing valuation. Previous studies have used structural equation modelling to examine the causal determinates of LS [63], and recent valuation studies have used this technique in valuing environmental goods [61].

In this study, a cross-lagged model was specified to examine the relationships among the variables of SRH, IS, HI, and LS. The full model analysed all the effects between the pairs of SRH, IS, HI and LS within and across the two-time points. In addition, the sample’s socio-demographic characteristics were hypothesised as covariates and were regressed on the main variable of interest (${\text{L}\text{S}}_{2}$). To check the model adequacy, the model’s comparative fit, parsimony correction, and absolute fit were assessed using the goodness-of-fit indices commonly used in the structural equation modelling technique, including the comparative fit index and Tucker-Lewis index, the Root Mean Square Error of Approximation, and Root Mean Square Residual. According to Hu and Bentler’s principle [64], the model is considered as having an adequate fit with the data when the Comparative Fit Index and Tucker-Lewis Index are > 0.90, the Root Mean Square Error of Approximation < 0.06, and Root Mean Square Residual < 0.08.

For the structural equation modelling estimation, data merged across the two waves of the survey was used for model fitting. Socio-demographic characteristics of the sample and their scores of SRH, IS, and LS across both waves are reported in Table 1. In comparison to population CENSUS [65], the sample retained in this survey were in general, older (i.e., 41.6% of the sample in this survey were aged 60 or above compared to 22.6% of the population were aged 60 or above in CENSUS) and consisted a greater proportion of individuals either were unemployed (i.e., 37.6% in this survey compared to 3.4% in CENSUS) and being economically inactive (i.e., 21.1% in this survey compared to 19.1% in CENSUS). In light of this, a weighting variable was constructed based on the sample’s age (i.e., 10-year age band) and employment status and was included in the cross-lagged analysis in order to reduce the potential bias due to the skewness of the sample [66]. In addition, samples’ equivalised monthly HI was derived from dividing their monthly HI by the square root of the household size. Also, the second wave sample’s monthly HI was deflated by the consumer price index for a valid reflection of the real changes in HI. Following convention, standardised coefficients of the cross-lagged model in structural equation modelling were reported.

**Table 1:** Sample’s socioeconomic characteristics and descriptive statistics on the main variables of interest (*N* = 1,109)

Sample’s characteristics	N (%)		
**Socioeconomic Status**			
**Age**	54.4 ± 16.9		
Sex			
Men	532 (47.9%)		
Women	577 (52.1%)		
**Employment status**			
Employed	458 (41.3%)		
Unemployed	417 (37.6%)		
Economically inactive	234 (21.1%)		
**Marital Status**			
Never married	219 (19.8%)		
Married / cohabited	677 (61.0%)		
Separated / divorced / Widowed	213 (19.2%)		
**Education attainment**			
Not educated	320 (28.9%)		
Primary	244 (22.0%)		
Secondary	348 (31.3%)		
Territory or above	197 (17.8%)		
**Household Size**			
One-person household	157 (14.1%)		
Two-person household	293 (26.4%)		
Three-person household	296 (26.7%)		
Four-person household or more)	363 (32.7%)		
**Main variables of interest**	**Wave 1**	**Wave 2**	
* SRH*	2.21 ± 0.91	2.20 ± 0.89	
* IS*	7.41 ± 2.69	7.59 ± 2.56	
* HI*	14.2 ± 13.0	16.5 ± 16.4	
* LS*	21.95 ± 7.23	21.95 ± 7.24	

*SRH *self-rated health status, *IS instrumental* support, *HI *equivalised monthly household income in thousand HK$; *LS *life satisfaction

Range of *SRH*: 1–5; range of *IS*: 3–12; range of *LS*: 5–35

Table 1 Sample’s socioeconomic characteristics and descriptive statistics on the main variables of interest (N = 1,109).

Building upon the findings from the cross-lagged analysis, the second stage of the valuation procedure was to estimate the willingness-to-pay of SRH and IS on LS. Specifically, the estimated causal effects (𝛽c) of ${\text{S}\text{R}\text{H}}_{1}$ and ${\text{I}\text{S}}_{1}$ on ${\text{L}\text{S}}_{2}$ as well as ${\text{H}\text{I}}_{1}$ on ${\text{L}\text{S}}_{2}$ were used for computing the marginal rate of substitutions of SRH and IS with respect to HI, respectively. Subsequently, it is substituted into the formulae of compensating surplus [38] expressed as,

$$\stackrel{-}{w}-{e}^{[{ln}\left(\stackrel{-}{w}\right)- \frac{{\beta }_{a}}{{\beta }_{b}}\varDelta x]}$$

where $\stackrel{-}{w}$ is sample’s median equivalised monthly HI, ${\beta }_{a}/{\beta }_{b}$ was the marginal rate of substitution, and $\varDelta x$ is the change in the non-market condition (i.e., SRH or IS). Estimates yielded from the formulae reflect the willingness to pay for the condition, which can be interpreted as (hypothetically) the amount of additional equivalised monthly HI required to equate the changes in LS in relation to the increase or decline either in SRH or IS.

## Results

### Cross-lagged analysis of the relationships between SRH, IS, HI, and LS

Using the structural equation modelling technique, the hypothesised cross-lagged model denoting the relationships among SRH, IS, HI, and LS, and the socio-demographic variables as covariates were examined. Model diagnostics suggested that there was an adequate fit between the specified model and the empirical data, as all the goodness-of-fit indices met the Hu and Bentler’s threshold (Comparative Fit Index = 0.91, Tucker-Lewis Index = 0.90, Root Mean Square Error of Approximation = 0.05, Root Mean Square Residual = 0.08). The measurement model of the latent variables were also adequate (IS: ranging from 0.703 to 0.769 in the baseline and from 0.738 to 0.774 in the follow-up, respectively; LS: ranging from 0.733 to 0.858 in the baseline and from 0.750 to 0.894 in the follow-up respectively). Table 2 summarises the findings of the cross-lagged analysis focusing on the relationships among SRH, IS, HI, and LS. The effects of the socio-demographic variables on samples’ LS are summarised in the appendix (see Additional file 3).^2^

**Table 2:** Standardized coefficients of the cross-lagged analysis on the relationships among *SRH, IS, HI*, and *LS*

Estimates of the cross-lagged analysis	Standardized Beta (95%CI)	
Temporal autoregressive effects		
* SRH*_*1*_ *→ SRH*_*2*_	0.587 (0.538, 0.637)***	
* IV*_*1*_ *→ IV*_*2*_	0.579 (0.464, 0.695) ***	
* HI*_*1*_ *→ HI*_*2*_	0.608 (0.565, 0.651) ***	
* LS*_*1*_ *→ LS*_*2*_	0.673 (0.563, 0.782)***	
Cross-sectional correlations		
*Baseline (1st -wave)*		
* SRH*_*1*_ ↔ *LS*_*1*_	0.319 (0.257, 0.380)***	
* IS*_*1*_ *↔ LS*_*1*_	0.524 (0.465, 0.584)***	
* HI*_*1*_ ↔ *LS*_*1*_	0.339 (0.563, 0.782)***	
*Follow-up (2nd -wave)*		
* SRH*_*2*_ ↔ *LS*_*2*_	0.135 (0.061, 0.210)***	
* IS*_*2*_ *↔ LS*_*2*_	0.229 (0.138, 0.320)***	
* HI*_*2*_ ↔ *LS*_*2*_	0.081 (0.005, 0.156)*	
Causal and Reverse Causal effects		
*Self-rated health and life satisfaction*		
* SRH*_*1*_ *→ LS*_*2*_	0.074 (0.021, 0.127)**	
* LS*_*1*_*→ SRH*_*2*_	0.086 (0.020, 0.151)*	
*Instrumental Support and life satisfaction*		
* IV*_*1*_ *→ LS*_*2*_	0.107 (0.042, 0.171)*	
* LS*_*1*_ *→ IV*_*2*_	− 0.008 (-0.084, 0.069)	
*Household Income and life satisfaction*		
* HI*_*1*_ *→ LS*_*2*_	0.073 (0.010, 0.137)*	
* LS*_*1*_ *→ HI*_*2*_	0.015 (-0.042, 0.123)	

*p < .05; **p < .01; ***p < .001

*SRH *self-rated health status, *IS *instrumental support, *HI *household income, *LS *life satisfaction

1: baseline; 2: follow-up

Table 2 illustrates that there were strong temporal autoregressive associations among the four variables (e.g., the temporal autoregressive association of SRH was 0.587), indicating that the sample’s scores on SRH, IS, LS, and HI at follow-up were strongly influenced by their respective scores in the baseline. The model also indicated that the sample’s SRH and LS were cross-sectionally associated with their satisfaction of life. Partially due to the adjustment of the temporal autoregressive associations, it is noted that the magnitude of these cross-sectional associations at follow-up were noticeably smaller than the respective estimates at baseline (e.g., the cross-sectional associations of SRH and LS were 0.319 for baseline versus 0.135 for the follow-up respectively). The cross-sectional associations between samples’ IS and LS and between HI and LS also displayed a similar pattern (SRH_1_ ↔ IS_1_: β = 0.231, 95% Confidence Interval: 0.162, 0.300; SRH_2_ ↔ IS_2_: β = 0.145, 95% Confidence Interval: 0.076, 0.214; SRH_1_ ↔ HI_1_: β = 0.159, 95% Confidence Interval: 0.095, 0.222; SRH_2_ ↔ HI_2_: β = 0.052, 95% Confidence Interval: -0.009, 0.13; SRH_2_ ↔ IS_*2*_: β = 0.145, 95% Confidence Interval: 0.076, 0.214; IS_1_ ↔ HI_1_: β = 0.080, 95% Confidence Interval: -0.002, 0.161; IS_2_ ↔ HI_2_: β = 0.041, 95% Confidence Interval: -0.101, 0.183).

Table 2 also summarises the causal and reverse causal effects of SRH, IS, HI on LS. Consistent with the expectation, the casual effects of SRH and IV on LS were significant even when their reverse causal effects were taken into account. Specifically, one standard deviation increases in the sample’s score on SRH and IS were casually associated with a standard deviation increase in their LS by the unit of 0.074 (95% Confidence Interval: 0.021, 0.127) and 0.107 (95% Confidence Interval: 0.042, 0.171), respectively. Similarly, the model also indicated that the causal influence of HI on LS was significant. One standard deviation increase in the sample’s HI was casually associated with a standard deviation increase in their LS by the unit of 0.073 (95% Confidence Interval: 0.010, 0.137). However, its respective reverse causal effect was not detected.

Table 2 Standardised coefficients of the cross-lagged analysis on the relationships among SRH, IS, HI, and LS.

### The implicit willingness-to-pays of SRH and IS on LS

Based on the results of the cross-lagged analysis (Table 2), the marginal rate of substitution of SRH and IS on LS were estimated to be 1.01 and 1.47, respectively. This can be interpreted as respondents’ increase (decrease) in LS owing to their improvement (deterioration) in SRH and IS is equivalent to the effect of increasing (reducing) their HI by 1.01 and 1.47 units of standard deviation, respectively. Substituting it into the formulae of compensating surplus, the implicit willingness-to-pay of improving the sample’s SRH from “poor health” to “excellent health” and enhancing their IS from “little support” to “a lot of support” are thus equivalent to an additional increase in their equivalised monthly HI by US$1,536 and US$1,523 respectively.^3^

## Discussion

Extensive research has shown that multiple aspects of life, such as health status and social relationships, would influence individuals’ wellbeing [39, 43]. Also, many interventions use these two as mechanisms to improve individual welfare. Hence, deriving their implicit willingness-to-pay provides tools to estimate the cost-effectiveness of initiatives aiming to improve individuals’ wellbeing. To the best of the authors’ knowledge, this is the first to derive the implicit willing-to-pays of SRH status and IS on LS in predominantly Chinese society, hence providing socio-culturally attuned estimates. It extends the wellbeing valuation literature on health and social outcomes as it is one of the few conducted in an Asian context.

This research has implications for the practice of social impact evaluation. Specifically, the derived shadow price of SRH and IS provide ways to tie social impact evaluation with some forms of cost-benefit analysis (e.g., social return on investment), which is a tool where policymakers commonly rely on for decision making [67]. Currently, in the absence of culturally-attuned economic proxies, practice among social impact evaluators sometimes apply economic proxies derived from another socio-cultural context (e.g., Western settings) to monetise social non-market outcomes, despite the notable cultural difference on individuals’ evaluation on domains of life [68, 69]. This study explores a way to make socio-cultural embedded cost-impact assessment plausible in the Chinese context.

Findings of the cross-lagged analysis illustrated that the relationships among the examined variables are complex. First, the model reflected the existence of significant autoregressive correlations within each main variable of interest (i.e., SRH, IS, HI, and LS). This is fairly intuitive given the timeframe between the two data collection time points was not long (i.e., twelve months). In addition, cross-sectional correlations among the variables of interest were also detected (e.g., between SRH and IS). Furthermore, the model suggested that the reverse causal mechanisms among these variables were plausible. Particularly, the sample’s baseline LS was positively and casually associated with their SRH. These findings are in line with previous research suggesting that positive affect can improve the immune system and reduce a person’s susceptibility to illness [70, 71]. Taken together, these findings add to a large pool of the domains-of-life literature highlighting the intertwining nature among individuals’ various aspects of life [2]. Perhaps the most important findings of the cross-lagged model are that after taking into account these effects among these variables (i.e., autoregressive, cross-sectional, and reverse causal effects), the model also pointed towards the existence of causal influences of SRH, IS, and equivalised monthly HI on samples’ satisfaction with life. It provides a key basis for the subsequent wellbeing valuation exercise.

The existence of the reverse causal influences that existed in the relationship between the sample’s SRH and LS further affirms the need to attend to the endogeneity problem in the regression analysis in order to avoid an overestimation of the implicit monetary value of health and social outcomes [45, 46]. In addition, this study also shows collinearity likely exists between exogenous variables (i.e., social and health conditions). It serves as a reminder for researchers to attend to this problem in future wellbeing valuation studies. The cross-lagged approach simultaneously addresses the cross-sectional associations between the exogenous variables, and the reverse-causal influences could be a plausible way to minimise the endogeneity issue in this line of valuation work.

Based on the concept of compensating surplus, this study estimated that the implicit willingness-to-pays of improving the sample’s SRH from “poor health” to “excellent health” and enhancing their IS from “little support” to “a lot of support” is equivalent to an additional increase in their equivalised monthly HI by US$1,536 and US$1,523 respectively. The implicit monetary value of SRH and IS estimated in this study was 1.43 and 1.03 times of the sample’s equivalised monthly HI (i.e., US$1,830). At first glance, these estimates may seem large. However, when contrasting with the willingness-to-pays derived from earlier wellbeing valuation studies, these estimates seem to fall within an expected range. For instance, one study in the US found the willingness-to-pay of SRH were 1.53 times of the median annual HI [39], and another study in Thailand found the respective estimates was 1.01–1.03 times of the monthly per capita HI [43]. In fact, the willingness-to-pay of SRH estimated in this study was found to be noticeably smaller than studies that did not take into account the endogeneity issue. For instance, one study in the UK reported that the willingness-to-pay of SRH was 31 times the sample’s annual real HI per capita [40]. Given this study is the first to derive the implicit monetary values of SRH and IS on an individual’s LS in a predominantly Chinese society, it requires future studies to further validate its accuracy. In addition, as mentioned earlier, the sample of the panel survey consisted of a greater proportion of older adults and individuals who are either unemployed or economically inactive. Despite the analysis using a weighting technique to counterbalance the potential influences on the valuation estimate, readers should exercise caution when generalising the estimation to other sub-populations (e.g., younger-age group).

This study is not without limitations. First, it was secondary data analysis, and hence the selection of variables for model estimation was restricted by the original survey design. For instance, the panel survey only consisted of questions on one type of social support (i.e., IS). Earlier studies pointed out that emotional support from families and friends is also an important determinant of an individual’s satisfaction with life [17]. In traditional Asian cultures, where verbal expression of love is rare, financial and material provision can carry the meaning of love and sacrifice as a form of emotional support [72]. However, we acknowledge that the survey design of this study cannot address this question. It warrants future research to explicitly test the contribution of emotional support and estimate its implicit monetary value. Second, although it is aware that the causal relationship between SRH, IS, and LS may vary across age and sex [73], this study, however, did not perform a sub-demographic analysis owing to a limited power due to the restricted sample size. While the willingness-to-pay estimated in this study may reflect the implicit monetary value of SRH and IS among the general population, caution should be exercised when generalising it to particular sub-demographic groups. Further research is warranted to estimate the variation. Third, as in almost all wellbeing valuation analyses, it cannot rule out the possibility that endogeneity still existed in the estimates, as the problem can arise from other sources (e.g., measurement error) that may not be able to be controlled from a statistical standpoint. Also, it cannot rule out other potential biases arising from the attrition of samples across data collection time points. Compared to other panel surveys with similar contexts (HKSAR), the attrition rate of this study was comparable [63, 64, 74, 75]. Nonetheless, this study represents the first effort entering this understudied study area, and it warrants future work to verify these results.

## Conclusions

This study adopted the wellbeing valuation approach to monetising the impact of increasing individuals’ SRH status and IS on their overall satisfaction with life. It has practical implications for the use of social cost-benefit analysis in assessing wellbeing policy initiatives for the Chinese population.

## Supplementary Information

**Additional file 1.** A conceptual illustration of the implicit willingness-to-pay of self-rated health with respect to an individual’s household income.

**Additional file 2.** Implicit willingness-to-pay of self-rated health status and social support from wellbeing valuation studies in an Asian context.

**Additional file 3.** Standardised coefficients of the cross-lagged analysis on the relationships of the socio-demographic variables on LS at T2.

## Data Availability

The datasets used and/or analysed during the current study are available from the corresponding author on reasonable request.

## Abbreviations

SRH: Self-rated health
IS: Instrumental Support
LS: Life Satisfaction
HI: Household Income

## Footnotes

1 Hong Kong was divided into 412 District Constituency Areas under 18 administrative districts and each geographical unit has a population of around 17,000 people on average.

2 The full cross-lagged model specified 201 effect estimates from 37 variables. For the purpose of clarity and parsimony, Table 2 only summarized effect estimates which are useful for the subsequent wellbeing valuation exercise.

3 Estimation of the willingness-to-pay of SRH and IS were based on the formulae of compensating surplus. In this estimation, $$\stackrel{-}{w}$$ is the sample’s equivalised monthly *HI*: HK$14.2k (i.e., US$1,830). The estimates are presented in US$ using the conversion rate of US$1 = HK$7.76. SRH (“poor health” [1] to “excellent health” [5]): US1830- exp(ln(1830)-1.01* 4/ 2.21); IS (“little support” [3] to “a lot of support” [12]): US1830- exp(ln(1830)-1.47 * 9/ 7.41).
