# Supplementary material for: Valuing the impact of self-rated health and instrumental support on life satisfaction among the chinese population
Source: BMC Public Health. 2022 Jun 20;22:1227. doi: 10.1186/s12889-022-13626-7 (PMC9210652; doi:10.1186/s12889-022-13626-7)
Supplement: Supplementary file 1 — Additional file 1. A conceptual illustration of the implicit willingness-to-pay of self-rated health with respect to an individual’s household income. [file 12889_2022_13626_MOESM1_ESM.pptx]

## Slide 1
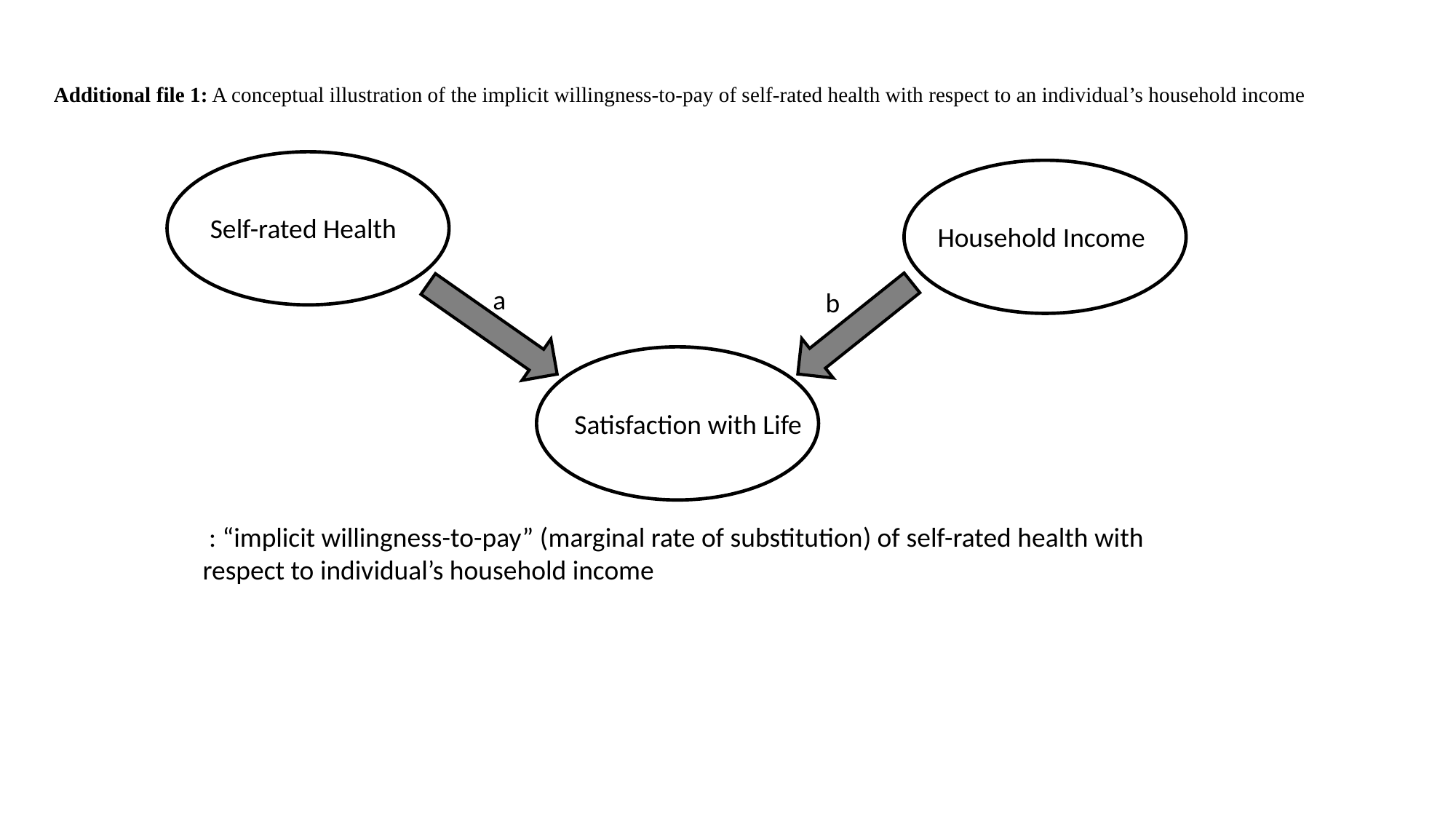

Additional file 1: A conceptual illustration of the implicit willingness-to-pay of self-rated health with respect to an individual’s household income
Self-rated Health
Household Income
a
b
Satisfaction with Life
