# Supplementary material for: Valuing the impact of self-rated health and instrumental support on life satisfaction among the chinese population
Source: BMC Public Health. 2022 Jun 20;22:1227. doi: 10.1186/s12889-022-13626-7 (PMC9210652; doi:10.1186/s12889-022-13626-7)
Supplement: Supplementary file 2 — Additional file 2. Implicit willingness-to-pay of self-rated health status and social support from wellbeing valuation studies in an Asian context. [file 12889_2022_13626_MOESM2_ESM.docx]

**Additional file 2:** Implicit WTPs of self-rated health status and instrumental support from well-being valuation studies in an Asian context

| **Context [study]** | **Source of Data** | **Adjustment of the endogeneity concern** | **Implicit**  **WTP** ^a,b^  (unit) | **Implicit WTP/**  **Corresponding HI** |
| --- | --- | --- | --- | --- |
| ***Self-rated health status*** | | | |  |
| United Kingdom, [Powdthavee, 2007] | British Household Panel Survey (BHPS), 1997-2003 | No | US$37,875 – US$38,000 (capita real HI) | 30.92-31.02 |
| United States,  [Brown, 2015] | Twenty-eight cross-sections of General Social Survey, 1975-2010 | Yes | US$3,471  (equivalised income) | 1.54 |
| Thailand  [Chandoevwit & Thampanishvong, 2016] | National cross-sectional survey, 2012 | Yes | US$137-US$141  (capita *HI*) | 1.01-1.03 |
| Japan  [Tsurumi & Managi, 2017] | National cross-sectional survey, 2013 | No | US$52,500  (*HI*) | 13.75 |
| ***Social Support / Social Interaction*** | | | |  |
| United Kingdom, [Powdthavee, 2007] | British Household Panel Survey (BHPS), 1997-2003 | No | *Meeting friends and relatives*  US$7,800- US$7,919  (capita *HI*)  *Talking to neighbours*  US$4,916 –US$5,100  (capita *HI*) | 6.37-6.51  4.01-4.16 |
| Thailand  [Chandoevwit & Thampanishvong, 2016] | National cross-sectional survey, 2012 | Yes | *Interaction with neighbours ^c^*  US$68  (capita *HI*) | 0.50 |
| Japan  [Tsurumi & Managi, 2017] | National cross-sectional survey, 2013 | No | *One-person increase in “reliable people”*  US$328 - US$679 (HI) | 0.09-0.18 |

^a^ Estimates of the WTPs are all standardized into $US based on the conversion rates reported within each study. In addition, all estimates are standardized on a monthly basis.

^b^ Responses ranges from “poor health” to “excellent health.”

^c^ Responses ranges from ^“^never” to “on most days”;

^d^ Responses ranges from ^“^no interactions” to “frequently”;

*HI:* household income; *Implicit WTP*: implicit willingness-to-pay
