# Supplementary material for: Valuing the impact of self-rated health and instrumental support on life satisfaction among the chinese population
Source: BMC Public Health. 2022 Jun 20;22:1227. doi: 10.1186/s12889-022-13626-7 (PMC9210652; doi:10.1186/s12889-022-13626-7)
Supplement: Supplementary file 3 — Additional file 3. Standardised coefficients of the cross-lagged analysis on the relationships of the socio-demographic variables on LS at T2. [file 12889_2022_13626_MOESM3_ESM.docx]

**Additional file 3:** Standardized coefficients of the cross-lagged analysis on the relationships of the socio-demographic variables on LS at T2

| Sample’s characteristics | **Beta (95%CI)** | |
| --- | --- | --- |
| **Socioeconomic Status** |  | |
| **Age** | 0.154 (-0.689, 0.997) | |
| Sex |  |  |
| Men | - |  |
| Women | 0.065 | (0.010, 0.120)* |
| **Employment status** |  |  |
| Employed | - |  |
| Unemployed | -0.038 | (-0.094, 0.018) |
| Economically inactive | -0.002 | (-0.071, 0.067) |
| **Marital Status** |  |  |
| Never married | - |  |
| Married/cohabited | -0.037 | (-0.115, 0.041) |
| Separated / divorced / Widowed | -0.059 | (-0.136, 0.017) |
| **Education attainment** |  |  |
| Not educated | - |  |
| Primary | 0.040 | (-0.025, 0.106) |
| Secondary | -0.022 | (-0.094, 0.050) |
| Territory or above | -0.013 | (-0.090, 0.065) |
| **Household Size** |  |  |
| One-person household | - |  |
| Two-person household | 0.065 | (-0.029, 0.159) |
| Three-person household | 0.055 | (-0.043, 0.154) |
| Four-person household or more) | -0.007 | (-0.112, 0.097) |

*LS:* life satisfaction

*T2: follow-up*
